# The Foreign Journals: Materia Medica

**Published:** 1839-04

**Authors:** 


### MATERIA MEDICA.

*Medicinal Properties of the Gray Oxide of Zinc.* By Professor SEMENTINI.

FROM a series of experiments made on the white and gray oxides of zinc (the latter discovered by himself) Professor Sementini, of Naples, draws the following conclusions: 1. The oxide of zinc possesses tonic properties, which it derives from its soothing the irritability of the nervous system; it is also antispasmodic and sedative. 2. This has long been known, but the use of the medicine has been abandoned, from the inconstancy of its effects. 3. That inconstancy arises from the facility with which it absorbs carbonic acid, and hence passes to the state of a subsalt. 4. The *gray* oxide does not absorb the acid, and is therefore always of uniform strength. 5. As the properties of a tonic and a sedative coexist in it, it may be used with the greatest confidence in cases of *irritative debility*. The dose to begin with is from a fourth to half a grain, which may be increased to four or six grains, by an addition of a quarter of a grain every second day.

*Giornale dell. Sc. Med. Chir.* No. 45. *Marzo,* 1838.

